# Difluoromethylornithine (DFMO) Enhances the Cytotoxicity of PARP Inhibition in Ovarian Cancer Cells

**DOI:** 10.3390/medsci10020028

**Published:** 2022-05-26

**Authors:** Olivia El Naggar, Brenna Doyle, Kelsey Mariner, Susan K. Gilmour

**Affiliations:** Lankenau Institute for Medical Research, 100 Lancaster Avenue, Wynnewood, PA 19096, USA; olivia.elnaggar118@gmail.com (O.E.N.); brennakdoyle27@gmail.com (B.D.); kelseycmariner@gmail.com (K.M.)

**Keywords:** polyamines, difluoromethylornithine, rucaparib, PARP inhibition, ovarian cancer

## Abstract

Ovarian cancer accounts for 3% of the total cancers in women, yet it is the fifth leading cause of cancer deaths among women. The BRCA1/2 germline and somatic mutations confer a deficiency of the homologous recombination (HR) repair pathway. Inhibitors of poly (ADP-ribose) polymerase (PARP), another important component of DNA damage repair, are somewhat effective in BRCA1/2 mutant tumors. However, ovarian cancers often reacquire functional BRCA and develop resistance to PARP inhibitors. Polyamines have been reported to facilitate the DNA damage repair functions of PARP. Given the elevated levels of polyamines in tumors, we hypothesized that treatment with the polyamine synthesis inhibitor, α-difluoromethylornithine (DFMO), may enhance ovarian tumor sensitivity to the PARP inhibitor, rucaparib. In HR-competent ovarian cancer cell lines with varying sensitivities to rucaparib, we show that co-treatment with DFMO increases the sensitivity of ovarian cancer cells to rucaparib. Immunofluorescence assays demonstrated that, in the presence of hydrogen peroxide-induced DNA damage, DFMO strongly inhibits PARylation, increases DNA damage accumulation, and reduces cell viability in both HR-competent and deficient cell lines. In vitro viability assays show that DFMO and rucaparib cotreatment significantly enhances the cytotoxicity of the chemotherapeutic agent, cisplatin. These results suggest that DFMO may be a useful adjunct chemotherapeutic to improve the anti-tumor efficacy of PARP inhibitors in treating ovarian cancer.

## 1. Introduction

Ovarian cancer is the leading cause of death from gynecological cancers in the United States, with an incidence rate of approximately 22,000 and 14,000 deaths every year [1]. Although ovarian cancer accounts for only 3% of all cancer in women, it is the fifth leading cause of cancer-related deaths for women in the United States due to difficulties in detecting this disease at the early stages and the challenges in treating advanced disease [1,2]. Ovarian cancers are generally sensitive to chemotherapy, and they often initially respond well to standard primary treatment with surgery and first-line platinum and taxane-based chemotherapy [3,4]. However, most patients experience recurrence of their cancer within 12–18 months [2]. Given that many high-grade ovarian cancers have defects in their DNA repair pathways, platinum-based therapies that induce double-strand DNA (dsDNA) breaks in tumor cells can result in tumor cell death and tumor debulking [5,6,7]. The main repair pathway for dsDNA breaks is homologous recombination (HR) repair, which is especially important in repairing collapsed replication forks during DNA replication [8]. Two of the most well-known HR proteins are BRCA1 and BRCA2 (breast cancer genes 1 and 2). BRCA1/2 mutations are found in most hereditary ovarian cancers with about 15% of women with high grade serous ovarian cancer (HGSOC) having a germline BRCA mutation and 5% having somatic BRCA mutations [9]. The Cancer Genome Atlas consortium has reported that up to 50% of patients with HGSOC harbor genetic lesions that confer HR deficiencies including BRCA1/2 mutations, BRCA1 methylation, or gene alterations in EMSY, phosphatase and tensin homolog (PTEN), ataxia telangiectasia mutated (ATM), Rad3-related protein (ATR), RAD51, or Fanconi-anemia-related gene (sometimes referred to as ‘BRCAness’) [10,11].

Another DNA repair enzyme is poly (ADP-ribose) polymerase (PARP), which recognizes single strand DNA (ssDNA) breaks and participates in DNA repair through the base excisional pathway by attaching DNA polymerase β to DNA break sites to replace missing bases [12,13]. PARP inhibition allows ssDNA breaks to persist, resulting in the conversion of ssDNA breaks to dsDNA breaks during DNA replication that must be repaired by HR to avoid cell death. In a synthetic lethal interaction, the targeted therapy of BRCA1/2-mutant ovarian cancer has been achieved using PARP inhibitors [14], since these tumors are deficient for the repair of dsDNA breaks by HR repair pathways [15]. Although the use of PARP inhibitors can significantly improve progression-free survival in patients with BRCA mutations, this has not translated to an overall survival benefit [3]. In ovarian tumors, a functional HR system and intact BRCA1/2 function are often associated with resistance to platinum-based chemotherapy and PARP inhibitors [16]. In addition, de novo and acquired resistance mechanisms further limit the efficacy of PARP inhibitors [17]. Many patients who initially respond to PARP inhibitors rapidly develop resistance and experience tumor progression [18]. Thus, it is important to identify the molecular mechanisms that contribute to platinum and PARP inhibitor resistance in ovarian cancer to better prevent tumor recurrences and improve overall survival.

Polyamines are dramatically elevated in tumor cells compared to normal cells due, in large part, to the induction of polyamine biosynthesis and uptake mechanisms by oncogenes such as c-MYC and RAS [19,20,21,22]. Polyamines (putrescine, spermidine, and spermine) are amino acid-derived polycations that play a major role in many functions in the cell. Some of these functions include DNA repair, as polyamines are also an essential part of the chromatin structure due to their polycationic properties [23]. Polyamines have been shown to stimulate the enzymatic activity and DNA binding of PARP1 [24,25] and to facilitate the association of HR repair proteins to damaged DNA [26]. Given this, elevated levels of polyamines may be contributing to the development of resistance to DNA-damaging platinum-based chemotherapy and may be an attractive target to augment existing anti-tumor therapies. We hypothesized that the inhibition of a key enzyme in polyamine biosynthesis with α-difluoromethylornithine (DFMO) will disrupt the DNA repair function of tumor cells and improve the efficacy of PARP inhibitors in HR-competent ovarian cancer cells. DFMO is an FDA-approved drug that specifically inhibits ornithine decarboxylase (ODC), the rate-limiting enzyme in polyamine biosynthesis [20]. DFMO has gained extensive data in the clinic and has been found to be safe and tolerated at high doses in children over several years as a part of a recent phase 2 neuroblastoma trial. Here, we show that DFMO treatment of ovarian tumor cell lines resulted in increased cisplatin-mediated cytotoxicity in HR-competent cells when combined with the widely used PARP inhibitor, rucaparib.

## 2. Materials and Methods

### 2.1. Cell Culture

All cells were obtained from the ATCC and were cultured in 5% CO_2_, 37 degrees. UWB1.289 parental cells and UWB1.289+BRCA cells were maintained in RPMI 1640 (Corning, Manassas, VA, USA, 10-040-CV)/MEGM (Lonza, Walkersville, MD, USA, CC-3150) basal medium supplemented with 3% FBS and 100 U/mL penicillin/streptomycin. OVCAR3 cells were maintained in RPMI 1640 (Corning, 10-040-CV) basal medium supplemented with 20% FBS, 0.01 mg/mL bovine insulin, and 100 U/mL penicillin/streptomycin. OVCAR5 and OVCAR8 cells were maintained in RPMI 1640 (Corning, Manassas, VA, USA, 10-040-CV) basal medium supplemented with 10% FBS and 100 U/mL penicillin/streptomycin. SKOV3 cells were maintained in McCoy’s 5A (ATCC^®^ 30-2007™) basal medium supplemented with 10% FBS and 100 U/mL penicillin/streptomycin.

### 2.2. Cell Viability Assays

Cells were plated in complete media in 96-well plates. The media was removed 24 h later, and the cells were refed with complete media ± 0.5 mM DFMO and/or 1, 5, 10, or 25 µM rucaparib. Then, 72 h after treatment, cell viability was assessed using the EZQuant Cell Quantifying Kit (Alstem, Richmond, CA, USA, CQ01) according to the manufacturer’s protocol. In other experiments, the cells were pretreated with DFMO for 24 h before pulsing cells with either H_2_O_2_ or cisplatin, followed by refeeding with complete media ± DFMO and/or rucaparib. Viability was assessed at varying times using the CellTiter-Glo Luminescent cell viability assay (Promega, Madison, WI, USA, G7572).

### 2.3. PARP Assay

Ovarian cancer cells were plated in complete media. The cells were in culture for 72 h prior to PAR quantification. UWB1.289 and UWB1.289+BRCA1 cells were pretreated with DFMO prior to being pulsed with H_2_O_2_. PAR was quantified using the PARP In Vivo Pharmacodynamic Assay II (Trevigen, Minneapolis, MN, USA, 4520-096-K) and normalized to protein.

### 2.4. Western Blot Analysis

Whole cell lysates were prepared in Pierce™ RIPA Lysis and Extraction Buffer (Thermo Scientific, Rockford, IL, USA, PI89900) and Halt™ Protease and Phosphatase Inhibitor Cocktail (Thermo Scientific, Rockford, IL, USA, PI78441). Proteins were separated by SDS page, transferred to PVDF membranes (Novex, Littleton, CO, USA, LC2002), and stained with Ponceau S. The membranes were blocked in 5% nonfat dry milk, followed by overnight incubation at 4 degrees with primary antibodies against γH2AX (Cell Signaling Technology, Beverly, MA, USA, 9718S), pS516 CHK2 (Cell Signaling Technology, Beverly, MA, USA, 2669S), pT68 CHK2 (Cell Signaling Technology, Beverly, MA, USA, 2661S), or pT284 XRCC1 (Invitrogen, Carlsbad, CA, USA, PA5-64861). Following primary antibody incubation, the membranes were incubated with an HRP-conjugated secondary antibody and imaged using the Pierce™ ECL 2 Western Blotting Substrate (Thermo Scientific, Rockford, IL, USA, PI80196) according to the manufacturer’s protocol. The membranes were probed with either α-tubulin (Calbiochem, Burlington, MA, USA, CP06) or histone H3 (Cell Signaling Technology, Beverly, MA, USA, 9715S) as a loading control.

### 2.5. Immunofluorescence and Inverted Microscopy

Cells were seeded in chamber slides (Thermo Scientific, Rockford, IL, USA, 12-565-8) in complete media ± DFMO. After DFMO pretreatment, the cells were pretreated with rucaparib for 30 min and then pulsed H_2_O_2_. The cells were fixed with 4% paraformaldehyde and permeabilized with 0.2% Triton. The cells were stained with either anti-PAR monoclonal (Sigma-Aldrich, clone 10H) or γH2AX (Cell Signaling Technology, Beverly, MA, USA, 9718S) primary antibodies, and then stained with either Cy™3-conjugated (Jackson ImmunoResearch, West Grove, NJ, USA, 115-165-003) or Alexa Fluor^®^488-conjugated (Jackson ImmunoResearch, West Grove, NJ, USA, 111-545-003) secondary antibodies. Slides were mounted with ProLong™ Gold Antifade Mountant with DAPI (Invitrogen, Carlsbad, CA, USA, P36941). The cells were visualized by fluorescence microscopy using a Zeiss Axiovert 200M inverted microscope. Images were captured using the AxioVision SE64 Rel. 4.9.1 software (Carl Zeiss Microscopy, White Plains, NY, USA), and then quantified using ImageJ 1.X software. The integrated density was recorded for each channel as a measure of fluorescence and was normalized to DAPI. At least three representative images were captured per treatment group.

### 2.6. PKH26 Staining to Detect Slow Cycling Cells

The cell membranes were labeled using the PKH26 Red Fluorescent Cell Linker kit (Sigma-Aldrich, Burlington, MA, USA, MIDI26-1KT) according to the manufacturer’s protocol. After 72 h in culture, the cells were stained with a viability dye and FLOW cytometry analysis was carried out using the BD FACSCanto II cytometer and analyzed using the FACSDiva Version 8.0.1 software (BD Biosciences, Franklin Lakes, NJ, USA).

### 2.7. Spermidine Uptake

The cells were plated in complete media and then treated with DFMO. Forty-eight hours after DFMO treatment, the cells were pulsed with ^3^H-spermidine for 1 h. The cells were washed with cold spermidine and were lysed with 0.1% SDS. The cell lysates were combined with scintillation fluid and analyzed using the Packard TopCount NXT scintillation counter (Packard, Meriden, CT, USA), and then normalized to protein.

### 2.8. Statistical Analysis

The data are represented as mean ± standard deviation (SD). Analyses were conducted using either a one-way ANOVA or a two-way ANOVA with a Tukey test to assess the statistical significance among groups using GraphPad Prism software v8 (GraphPad Software, Inc., La Jolla, CA, USA). All data points refer to the technical repeats. Statistical significance was considered at test level *p* ≤ 0.05.

## 3. Results

### 3.1. DFMO Sensitizes HR-Competent Ovarian Cancer Cells to Rucaparib

**Figure 1:**
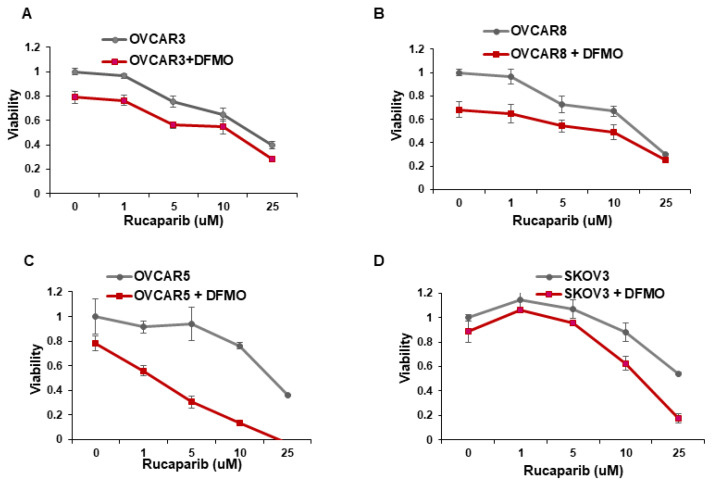
*DFMO sensitizes ovarian tumor cell lines to rucaparib treatment.* (**A**–**D**) OVCAR3, OVCAR8, OVCAR5, and SKOV3 were treated with 0.5 mM DFMO and increasing concentrations of rucaparib (1, 5, 10, 25 µM). Viability was assessed 72 h later via an MTS assay.

Whereas ovarian cancer cells with BRCA1/2 mutations are sensitive to the PARP inhibitor rucaparib [14,15], multiple cell lines with wild-type BRCA1/2 show varying sensitivity to rucaparib [27]. We investigated whether the inhibition of the polyamine biosynthetic pathway with DFMO can increase rucaparib sensitivity of four wildtype BRCA1/2 ovarian cancer cell lines. Cell viability was assessed over a wide range of rucaparib concentrations (1–25 µM) in the absence or presence of DFMO. In agreement with previous studies [27], the OVCAR5 and SKOV3 cells were relatively resistant to rucaparib compared to the more sensitive OVCAR3 and OVCAR8 cells (Figure 1). Likewise, while 0.5 mM DFMO treatment alone had no significant effect on viability in OVCAR5 and SKOV3 cells, 0.5 mM DFMO reduced the viability in OVCAR3 and in OVCAR8 cells (Figure 1). Interestingly, 0.5 mM DFMO sensitized the rucaparib-resistant OVCAR5 and SKOV3 cells to rucaparib, particularly at higher concentrations (10–25 µM) of rucaparib (Figure 1).

Increasing evidence shows that resistance to chemotherapeutic agents is often mediated by a subpopulation of slow-cycling cancer stem cells that display increased efficiency in DNA damage repair [28,29,30]. Using the fluorescent membrane dye PKH26 to characterize the percentage of slow-cycling cells [31], SKOV3 cells retained the most PKH26 fluorescence after 72 h in culture, indicating a slow-cycling phenotype (Supplemental Figure S1). On the other hand, OVCAR3 and OVCAR8 retained less PKH26 dye, indicating a lower percentage of slow-cycling cells and also correlating with their increased sensitivity to rucaparib (Figure 1A,B).

### 3.2. DFMO Reduces PARP-Mediated PARylation in the Presence of H_2_O_2_

**Figure 2:**
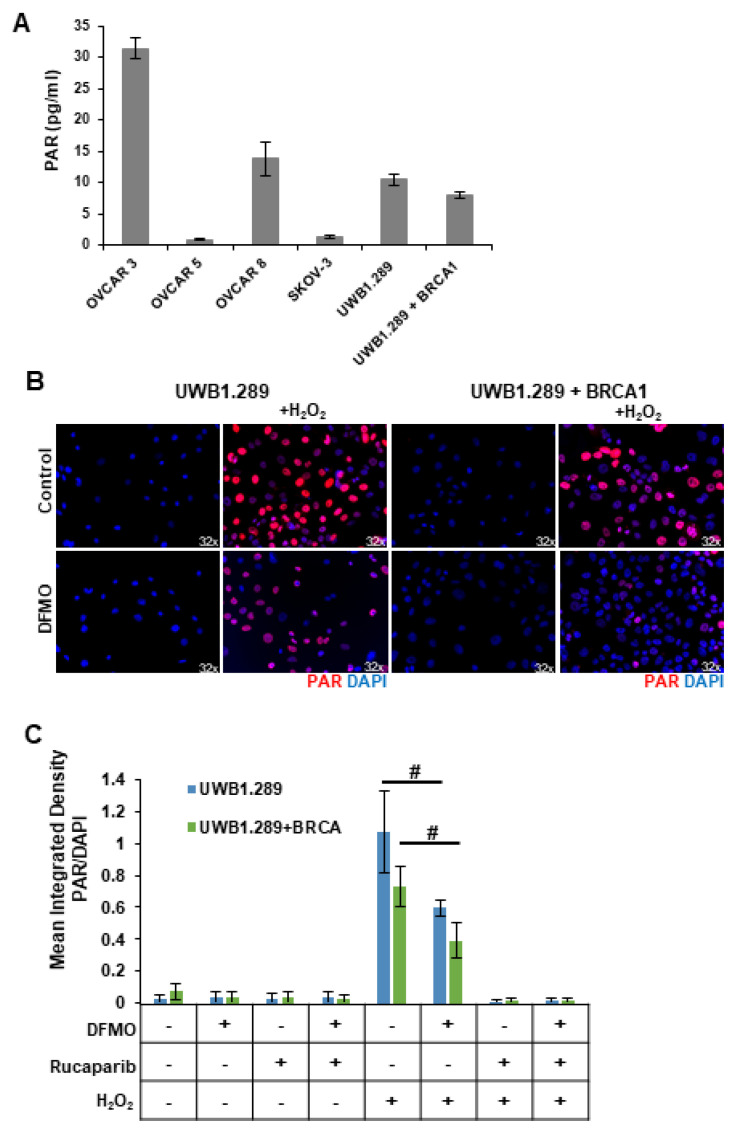
*DFMO treatment reduces H_2_O_2_-induced PARylation in ovarian tumor cell lines.* (**A**) Basal levels of poly (ADP-ribose) (PAR) were measured in OVCAR3, OVCAR5, OVCAR8, SKOV3, UWB1.289, and UWB1.289+BRCA after 72 h in culture. (**B**) UWB1.289 and UWB1.289+BRCA cells were pretreated with 1 mM DFMO for 24 h prior to a 10 min treatment with 4 mM H_2_O_2_. PARP activity was assessed via immunofluorescence in fixed cells 10 min after H_2_O_2_ treatment was removed. Nuclei were stained with DAPI. (**C**) Mean integrated density of PAR/DAPI fluorescence after H_2_O_2_ treatment. # *p* ≤ 0.01.

Despite their BRCA-wildtype status, HR-competent cells rely on PARP-mediated DNA repair with varying dependency. Because intracellular polyADP-ribose (PAR) levels may represent an important marker for how an ovarian tumor will respond to a PARP inhibitor, we compared basal PARP activity in a panel of ovarian cell lines using a chemiluminescent ELISA assay to quantitate the PAR levels in the cellular extracts. The rucaparib-sensitive OVCAR3 and OVCAR8 cells showed the highest basal levels of PAR, while SKOV3 and OVCAR5 cells, which are more resistant to rucaparib, correlated with low basal levels of cellular PAR (Figure 2A). UWB1.289, a BRCA1-null ovarian cancer cell line, displayed slightly higher basal PAR levels compared to UWB1.289+BRCA cells, which have restored BRCA1 function (Figure 2A), perhaps reflecting greater reliance on PARP activity for DNA damage repair in the HR-deficient UWB1.289 parental cells.

Reactive oxygen species (ROS) are present in higher-than-normal concentrations in tumor cells, and ROS such as hydrogen peroxide (H_2_O_2_) cause single-strand breaks that are recognized by PARP [32,33]. Thus, we introduced H_2_O_2_ into in vitro cultures to provide a simplistic model of the DNA-damaging ROS present within the tumor microenvironment. HR-deficient UWB1.289 cells and HR-competent UWB1.289+BRCA were briefly pulsed with 4 mM H_2_O_2_. Given previous findings that polyamines can serve as cofactors for PARP [24,25], we investigated the effects of polyamine depletion with DFMO on basal and H_2_O_2_-induced PARP activity. Prior to H_2_O_2_ pulse, some cells were pretreated for 24 h with 1 mM DFMO or were treated with rucaparib for 30 min. Immunofluorescence staining with an anti-PAR antibody revealed a strong induction of poly-ribosylated proteins following H_2_O_2_ exposure (Figure 2B,C). Interestingly, DFMO treatment significantly decreased cellular PAR levels in both UWB1.289 and UWB1.289+BRCA cells (Figure 2B,C). As expected, the intracellular PAR levels were completely knocked down with rucaparib treatment (images not shown, Figure 2C). Similar results were obtained using an ELISA assay to quantitate PAR levels in cellular extracts (Supplemental Figure S2). These results suggest that DFMO disrupts PARylation in response to oxidative DNA damage, contributing to a reduced ability to repair DNA damage and an increased sensitivity to PARP inhibition in HR-competent ovarian cancer cells.

### 3.3. DFMO Increases DNA Damage Accumulation and Sensitizes HR-Competent Ovarian Cancer Cells to Rucaparib in Presence of H_2_O_2_

**Figure 3:**
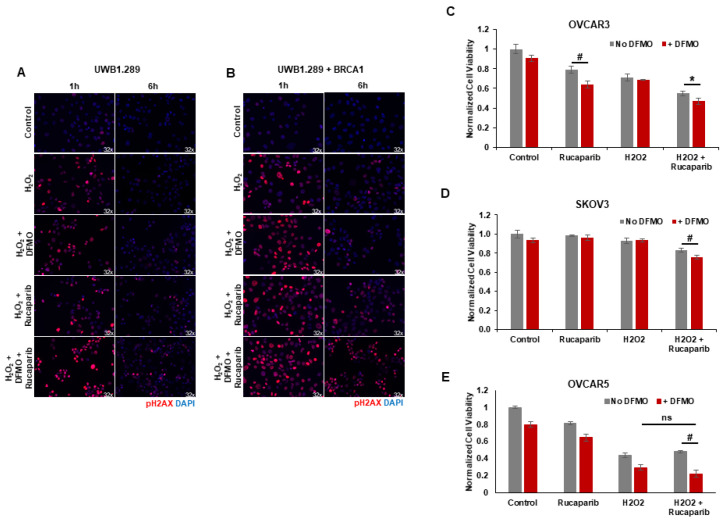
*DFMO and rucaparib treatment increases H_2_O_2_-induced DNA damage in ovarian tumor cell lines.* (**A**) UWB1.289 cells were pretreated with 1 mM DFMO for 24 h prior to treatment with 10 µM rucaparib and a 20 min pulse with 4 mM H_2_O_2_. DNA damage was assessed via immunofluorescence of γH2AX in fixed cells 1 h and 6 h after H_2_O_2_ treatment was removed. Nuclei were stained with DAPI. (**B**) UWB1.289 + BRCA cells were pretreated with 1 mM DFMO for 24 h prior to treatment with 10 µM rucaparib and a 20 min pulse with 4 mM H_2_O_2_. DNA damage was assessed via immunofluorescence of γH2AX in fixed cells 1 h and 6 h after H_2_O_2_ treatment was removed. Nuclei were stained with DAPI. (**C**) OVCAR-3 cells were pretreated with 1 mM DFMO for 24 h prior to treatment with 10 µM rucaparib and a 20 min pulse with 10 mM H_2_O_2_. Viability was assessed 24 h after H_2_O_2_ treatment, and all values are normalized to untreated control. Results represent the average of two experiments. (**D**) SKOV3 cells were pretreated with 0.1 mM DFMO for 24 h prior to treatment with 7 µM rucaparib and a 20 min pulse with 3 mM H_2_O_2_. Viability was assessed 48 h after H_2_O_2_ treatment, and all values are normalized to untreated control. (**E**) OVCAR5 cells were pretreated with 1 mM DFMO for 24 h prior to treatment with 10 µM rucaparib and a 5 h pulse with 100 µM H_2_O_2_. Viability was assessed 24 h after H_2_O_2_ treatment, and all values are normalized to untreated control. # *p* ≤ 0.01; * p < 0.05; ns: not significant.

Upon detection of DNA damage, histone H2AX is phosphorylated at serine 139 (γH2AX) by ATM at sites of dsDNA breaks or by ATR at ssDNA breaks [34]. H2AX remains phosphorylated until the damage is repaired [35,36,37]. We used γH2AX as a functional marker of DNA damage to analyze the effects of rucaparib and DFMO co-treatment on the accumulation of DNA damage in HR-competent UWB1.289+BRCA ovarian cancer cells and HR-deficient UWB1.289 cells. Following pretreatments with 1 mM DFMO for 24 h and 10 µM rucaparib for 30 min, the cells were pulsed with 4 mM H_2_O_2_ for 20 min and then harvested for at 1 h and 6 h following the H_2_O_2_ pulse. Immunofluorescence staining revealed an induction of nuclear γH2AX foci 1 h after H_2_O_2_ treatment in both UWB1.289 and UWB1.289+BRCA with increased γH2AX staining in cells treated with DFMO and rucaparib (Figure 3A,B). At 6 h after H_2_O_2_ treatment, much of the DNA damage (as measured by γH2AX foci staining) had resolved in both vehicle control and DFMO-treated UWB1.289 and UWB1.289+BRCA cells. Although significantly decreased by 6 h after H_2_O_2_ treatment, γH2AX foci were still detected in a small percentage of both UWB1.289 and UWB1.289+BRCA cells treated with rucaparib. However, co-treatment with DFMO and rucaparib significantly amplified the number of γH2AX foci, particularly in the HR-competent UWB1.289+BRCA cells, indicating increased DNA damage in these cells (Figure 3B). Similar results were observed with OVCAR3 cells, in which co-treatment with DFMO and rucaparib significantly increased DNA damage (γH2AX levels) in H_2_O_2_-exposed cells compared to that with vehicle control or rucaparib alone (Supplemental Figure S3).

Given that co-treatment with DFMO and rucaparib resulted in greater accumulation of DNA damage following the induction of ssDNA breaks with H_2_O_2_ exposure in HR-competent UWB1.289+BRCA and OVCAR3 cells, we assessed the effect of brief H_2_O_2_ exposure and treatment with DFMO and/or rucaparib on cell viability in HR-competent ovarian cancer cell lines possessing varying sensitivity to rucaparib. Following short-term exposure to H_2_O_2_, rucaparib treatment alone decreased the cell viability in OVCAR3 cells, but not in OVCAR5 or SKOV3 cells. However, co-treatment with DFMO significantly increased sensitivity to rucaparib with reduced cell viability in both OVCAR5 and SKOV3 cells following the induction of DNA damage with H_2_O_2_ (Figure 3C–E). These results indicate that DFMO intensifies the cytotoxic potential of the PARP inhibitor rucaparib via additional inhibitory effects on the DNA repair machinery.

### 3.4. DFMO Suppresses CHK2 and Downstream Events

**Figure 4:**
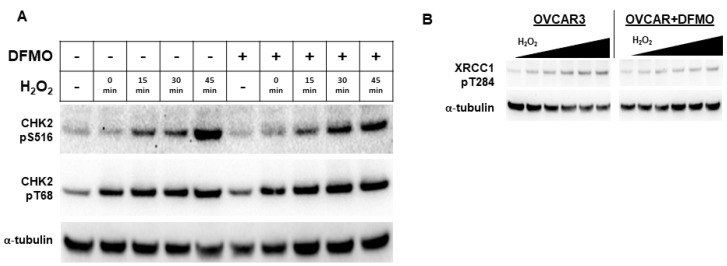
*DFMO suppresses CHK2 kinase function and XRCC1 in OVCAR3 cells.* (**A**) OVCAR3 cells were pretreated with 1 mM DFMO for 24 h and then treated with a 20 min pulse with 4 mM H_2_O_2_, followed by an incubation in drug-free or DFMO-containing media for up to 45 min. Cell lysates were probed for CHK2 phospho-serine 516, CHK2 phospho-threonine 68, and α-tubulin for a loading control in a Western blot assay. (**B**) OVCAR3 cells pretreated with 2 mM DFMO for 24 h prior to receiving 20 min treatments with varying concentrations of H_2_O_2_ show reduced XRCC1 T284 phosphorylation compared to controls. H_2_O_2_ was washed out and cells were incubated for 45 min before harvest with RIPA lysis buffer. Lysates were immunoblotted for XRCC1 pT284, and were normalized to the loading control, α-tubulin.

Because DFMO appears to interfere with PARP function, we examined an upstream kinase that has been reported to facilitate PARP function in the DNA repair response. Checkpoint kinase 2 (CHK2) is a central signaling protein in the DNA repair response, having functions that feed into both base excision repair (BER) and HR [38]. CHK2 phosphorylation of PARP facilitates the nuclear localization of PARP and enhances PARP activity [39]. As a DNA damage sensor and master regulator, ATM phosphorylates CHK2 at residue Thr68 (CHK2pT68), leading to activated CHK2 [38,40]. To determine whether the DFMO inhibition of polyamine biosynthesis may alter CHK2 activation that is an upstream regulator of PARP localization and activity, we examined the phosphorylation status of CHK2 in OVCAR3 cells following oxidative DNA damage. Following H_2_O_2_ exposure, OVCAR3 cells displayed an increase in CHK2pT68 with or without DFMO pretreatment (Figure 4A and Figure S4A). This suggests that the initial activation of CHK2 is unimpaired by DFMO. However, to activate downstream DNA repair proteins, CHK2 must have kinase functionality, which is measured by its autophosphorylation at residue Ser516 [41]. When probed for Ser516 phosphorylated CHK2, OVCAR-3 cells demonstrated a time-dependent increase in phosphorylation without DFMO, with a more than 7-fold induction over baseline at 45 min after H_2_O_2_ exposure. In contrast, the DFMO-pretreated cells showed a small increase in S516 phosphorylation over time, but phosphorylation was substantially lower than without DFMO treatment (Figure 4A). Taken together, these findings suggest that the activation of CHK2 is not affected by DFMO treatment, but DFMO may impair CHK2 kinase function and therefore suppress signaling in the DNA repair response.

Another downstream target of CHK2 is the BER scaffold protein, XRCC1. After activation, XRCC1 translocates to the sites of DNA damage marked by PAR, bringing with it repair proteins such as DNA ligase IIIα and DNA polymerase β [42]. CHK2 phosphorylates XRCC1 at the Thr284 residue (XRCC1pT284), enhancing its interaction with sites of DNA damage and promoting BER [43]. To determine whether DFMO affects XRCC1 phosphorylation, we treated OVCAR3 cells with DFMO and increasing concentrations of H_2_O_2_. Whereas H_2_O_2_ treatment increased levels of XRCC1pT284 in a dose-dependent manner, pretreatment with DFMO suppressed the induction of XRCC1pT284 following oxidative DNA damage (Figure 4B and Figure S4B). These results suggest that CHK2-mediated phosphorylation of XRCC1 is impaired by DFMO and may broadly suggest that optimal CHK2 kinase function depends on polyamines.

### 3.5. DFMO and Rucaparib Enhance Cytotoxicity of Cisplatin in HR-Competent Cell Lines

**Figure 5:**
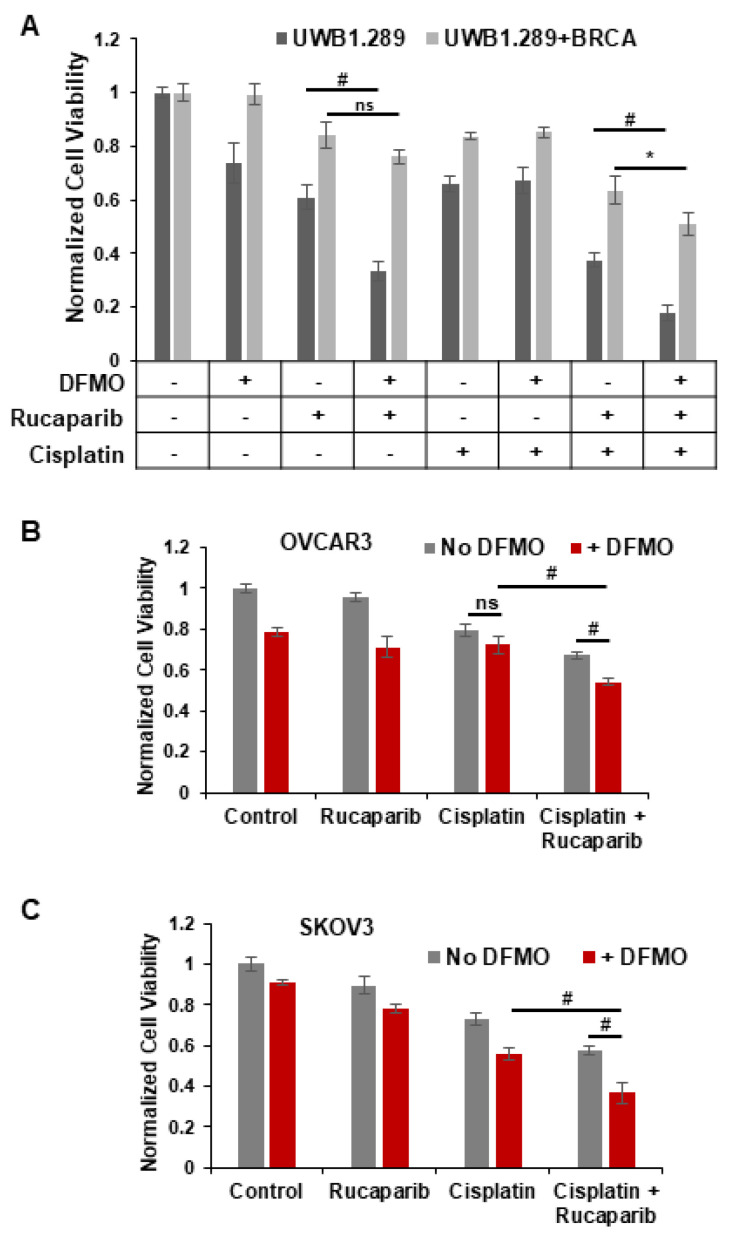
*DFMO and rucaparib treatment enhances cytotoxicity of cisplatin in ovarian tumor cell lines.* (**A**) UWB1.289 and UWB1.289+BRCA cells were pretreated with 0.2 mM DFMO for 24 h prior to treatment with 25 µM rucaparib and a 2 h pulse with 1.5 µg/mL (5 µM) cisplatin. Viability was assessed 72 h after cisplatin treatment. (**B**) OVCAR3 cells were pretreated with 1 mM DFMO for 40 h prior to treatment with 10 µM rucaparib and a 3 h pulse with 4 µg/mL cisplatin. Viability was assessed 72 h later. (**C**) SKOV3 cells were pretreated with 0.1 mM DFMO for 24 h prior to treatment with 7 µM rucaparib and a 2 h pulse with 3 µg/mL cisplatin. Viability was assessed 72 h after cisplatin treatment, and all values were normalized to untreated control. # *p* ≤ 0.01; * p < 0.05; ns: not significant.

Standard therapy for advanced ovarian cancer includes platinum-based chemotherapies that form bulky platinum-DNA adducts and lead to DNA crosslinks and dsDNA breaks in tumor cells [44]. Since HR is one of the major pathways for the repair of dsDNA breaks, PARP inhibitors are administered to patients who are deficient in HR-associated genes such as BRCA1/2. As expected, platinum sensitivity correlates with sensitivity to PARP inhibitors [27]. However, resistance to platinum therapy occurs in HR-competent ovarian cancer as well. We sought to discover whether the combination of rucaparib and DFMO acts as an effective enhancer of cisplatin sensitivity in HR-competent ovarian tumor cells. We analyzed the effects of rucaparib and/or DFMO on the cell viability in HR-deficient UWB1.289 and HR-competent UWB1.289+BRCA cells following a 2 h pulse of 1.5 µg/mL cisplatin. There were distinct differences between their sensitivity to rucaparib and DFMO treatment. Treatment with DFMO or rucaparib alone significantly decreased the viability of UWB1.289 cells, but not in their HR-competent counterpart, UWB1.289+BRCA (Figure 5A). Co-treatment of DFMO and rucaparib led to a significant reduction in viability compared to rucaparib alone in UWB1.289 cells, but not in UWB1.289+BRCA. As expected, cisplatin-induced DNA damage reduced viability in both cell lines. However, with the need to repair dsDNA breaks following cisplatin treatment, DFMO significantly increased the sensitivity of HR-competent UWB1.289+BRCA cells to rucaparib with decreased viability in both UWB1.289 and UWB1.289+BRCA cells (Figure 5A). Interestingly, polyamines have been shown to enhance DNA strand exchange activity of RAD51 recombinase in HR repair [26]. DFMO-increased sensitivity to rucaparib may be attributed, at least in part, to DFMO-reduction in polyamine biosynthesis that has been shown to interfere with HR repair. These data highlight the key differences between HR-deficient and HR-competent cells, but also show the ability of DFMO and rucaparib treatment to confer increased susceptibility of HR-competent cells to cisplatin.

We used the HR-competent cell lines SKOV3 and OVCAR3 to further investigate the combinatorial effects of DFMO, rucaparib, and cisplatin in ovarian cancer cells with wild-type BRCA1/2 functionality [45]. Without cisplatin-induced DNA damage, DFMO treatment with or without rucaparib had only a moderate effect on cell viability in both SKOV3 and OVCAR3 cells (Figure 5B,C). While cisplatin alone led to a significant decrease in viability, combined treatment with DFMO, rucaparib, and cisplatin showed the greatest decrease in viability, suggesting a cooperative effect from DFMO, rucaparib, and cisplatin on viability. Interestingly, following cisplatin treatment, DFMO sensitized rucaparib-resistant SKOV3 cells to rucaparib treatment with a clear additive effect of DFMO and rucaparib treatment to decrease viability (Figure 5C). These data demonstrate the cooperative effect of DFMO and rucaparib following the induction of dsDNA breaks with cisplatin.

## 4. Discussion

**Figure 6:**
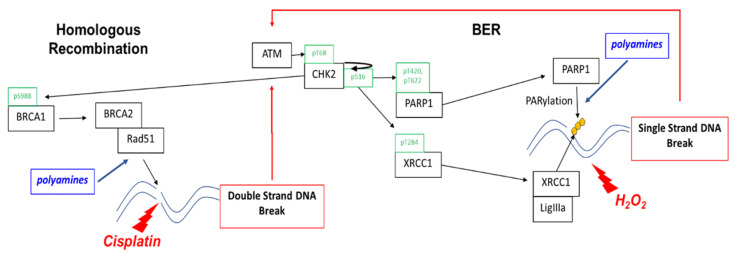
*Polyamines contribute to the repair of single-strand and double-strand breaks.* Homologous recombination enables cells to achieve high-fidelity repair of double-strand breaks and other complex DNA damage. PARP1 is essential for single-strand break (SSB) repair by binding to DNA at the sites of SSBs and recruiting repair machinery. PARP repair via the base excision repair (BER) pathway is the default repair pathway in HR deficient cells, such as that which occurs with loss of BRCA1 or BRAC2 function. As a DNA damage sensor and master regulator, ATM phosphorylates CHK2 at residue Thr68 followed by autophosphorylation at residue Ser516, activating CHK2 that phosphorylates proteins involved in both base excision repair and HR. CHK2 phosphorylation of PARP facilitates nuclear localization of PARP and enhances PARP activity. CHK2 also phosphorylates XRCC1 at the Thr284 residue, enhancing its interaction with sites of DNA damage and promoting base excision repair. Polyamines are PARP1 cofactors and enhance PARP activity in ssDNA break repair. Polyamines enhance DNA strand exchange activity of Rad51 recombinase in HR repair and may also contribute to PARylation of dsDNA break repair proteins.

This study shows that a low cytotoxic dose of DFMO significantly increases rucaparib-induced DNA damage and cytotoxicity in ovarian cancer cells regardless of HR repair status. DFMO acts in a multifaceted way to enhance the cytotoxic effects of PARP inhibitors, suggesting that it may be a useful adjunct chemotherapeutic approach to improve the anti-tumor efficacy of PARP inhibitors in treating ovarian cancer. Polyamines have roles in the repair of not only dsDNA breaks, but also ssDNA breaks by serving as a co-factor for PARP (Figure 6). Polyamines stimulate the enzymatic activity of PARP1 (via both a Mg^2+^-sparing effect and protection of the PARP enzyme from abortive binding to denatured DNA), and they facilitate PARP enzyme-DNA binding to assist in repair [24,25]. In addition to its direct role in PARylating sites of DNA damage, PARP has also been documented to PARylate and modify the function of numerous DNA repair proteins, including ATM and BRCA1 [12]. For instance, BRCA1 PARylation prevents dysfunctional HR repair and chromosomal rearrangements [46]. Considering our observation that DFMO affects PARP activity, it is possible that perturbing polyamine levels also alters the activity and function of ATM and BRCA1 by reducing PARylation on these key components of the DNA repair response. Moreover, polyamines are essential for the HR repair process via the enhancement of DNA strand exchange activity of RAD51 recombinase at sites of DNA damage [26]. Although we found that DFMO by itself only has a minor effect on the accumulation of DNA damage, DFMO coupled with PARP inhibition can overcome the DNA repair capacity of HR-competent ovarian tumor cells.

In our study, DFMO also alters the kinase activity of CHK2, which phosphorylates a wide array of proteins within the DNA-repair process. BRCA1 becomes phosphorylated by CHK2 in HR repair, enhancing the end resection process in the early stages of HR repair [47]. CHK2 also phosphorylates BRCA2, promoting the release of Rad51 from BRCA2 to engage DNA strands at the site of HR repair [48]. Besides the phosphorylating proteins involved in the HR repair response, CHK2 phosphorylates PARP1, driving nuclear localization and catalytic activity in response to oxidative DNA damage [39]. In addition, CHK2 phosphorylates the scaffold protein XRCC1, supporting its interaction with sites of DNA damage during base excision repair [43]. Because CHK2 has a central role in promoting DNA repair, the DFMO-mediated attenuation of CHK2 kinase activity has the potential to reduce the efficiency of downstream repair effectors and/or reduce the expression of key repair proteins that are involved in HR repair and base excision repair.

A common survival strategy in PARP inhibitor-resistant tumor cells is a restoration of BRCA1/2 function through secondary mutations or epigenetic de-repression of BRCA1 or BRCA2, thus restoring the high-fidelity repair of dsDNA breaks by HR DNA repair to escape the synthetic lethality caused by PARP inhibition [49,50]. Besides directly exerting stabilizing and activating effects on PARP, elevated intracellular polyamine levels in tumor cells increase histone acetyltransferase (HAT) activity [51,52,53,54,55] that, in turn, may increase the acetylation and activation of BRCA1WT [56]. It has been shown that pathways clustered around S-adenosylmethionine (SAM), functioning as a methyl donor for the methylation of histones, DNA, RNA, and for the biosynthesis of polyamines, are highly associated with resistance to cisplatin in ovarian cancer [57,58]. Metabolomics studies have revealed that the biosynthesis of polyamines, methionine, and glutathione represents the most significantly altered pathways associated with the development of platinum resistance in ovarian cancer [57,58]. Thus, increased polyamine levels in ovarian tumors can lead to chromatin remodeling involving acetylation and methylation of histones and DNA of specific genes to increase DNA repair and chemotherapy resistance in ovarian tumor cells.

Increased levels of the polyamine, spermidine, increase the activity of the translation factor eIF5A via post-translational modification with hypusine, which is derived from spermidine [59]. eIF5A has recently been shown to operate as a “translatome remodeler” that suppresses metabolism and prevents DNA damage in acidic microenvironments, where chemo-resistant stem-like tumor cells can reside [60]. Endogenous reactive oxygen species (ROS) levels are increased in slow-cycling cancer stem cells as a result of increased mitochondrial respiration and oxidative phosphorylation [61,62]. Because polyamines exert antioxidant functions by neutralizing free radicals and protecting DNA from oxidation [63], they play an essential role in the ability of tumor cells to adapt to hypoxic stress. Whereas ROS induce DNA damage, cancer stem cells display more efficient DNA repair, conferring resistance to treatment with PARP inhibitors [30,64,65]. In both mutant BRCA and wild-type BRCA ovarian tumors, cancer stem cells have been found to be resistant to PARP inhibition and to display increased RAD51 foci formation efficiency after DNA damage [30,66]. In part, this may be due to c-MYC and mutant K-RAS increasing HR-mediated DNA repair via the increased expression of RAD51, BRCA1/2, and MRE11 [67,68]. Indeed, high levels of oncogenic c-MYC are found in 65% of human ovarian cancers [10,69] and are associated with the activation of stem cell-like potential [70,71,72,73,74,75], faster recurrence, platinum resistance, and the poor overall survival of ovarian cancer patients [76,77]. It has been proposed that the acquired resistance to PARP inhibitors is due to the activation of HR DNA repair mechanisms that lead to more efficient DNA repair in cancer stem cell populations [66]. Since ODC transcription is upregulated by the oncogenes, MYC and RAS [21,22], the association of high levels of MYC and mutant RAS in ovarian tumor cells resistant to PARP inhibitors and platinum-based chemotherapy may be mediated, at least in part, by polyamines. Since polyamines are essential for efficient HR repair [26], a polyamine targeted therapy (such as DFMO with or without a polyamine transport inhibitor) would be predicted to enhance ovarian tumor sensitivity to a PARP inhibitor by impairing critical HR-mediated DNA repair function (upregulated with oncogenic c-MYC, RAS activation, and wild-type BRCA1/2). Our results demonstrate that co-treatment with DFMO and the PARP inhibitor, rucaparib, significantly enhances the accumulation of DNA damage, leading to cell death in ovarian tumor cells that are resistant to PARP inhibitors. From a clinical perspective, DFMO may be a useful adjunct chemotherapeutic approach to improve the anti-tumor efficacy of PARP inhibitors in treating ovarian cancer.

## Data Availability

Data supporting reported results are contained within this article.

## Supplementary Materials

The following are available online at https://www.mdpi.com/article/10.3390/medsci10020028/s1, Figure S1: Polyamine uptake correlates to a slow cycling characteristic in HR competent cell lines. (A) OVCAR3, OVCAR5, OVCAR8, and SKOV3 cells were stained with red PKH26 fluorescent cell membrane dye and analyzed via FLOW cytometry after 72 h in culture. (B) OVCAR3, OVCAR5, OVCAR8, and SKOV3 cells were treated with 1 mM DFMO. Spermidine uptake was analyzed 48 h later as a measure of polyamine transport activity. Data are represented as fold induction of polyamine transport activity. Figure S2: DFMO reduces PARylation in both HR-competent and HR-deficient cells. (A) UWB1.289 and UWB1.289+BRCA1 cells were pretreated with 3 mM DFMO for 26 h prior to treatment with 1 mM H_2_O_2_ for 20 min. H_2_O_2_ was washed out and cells were incubated for 30 min before lysis. PAR levels were quantified using Trevigen HT PARP in vivo pharmacodynamics kit. Figure S3: DFMO and rucaparib increase γH2AX in the presence of H_2_O_2_ in OVCAR3 cells. (A) OVCAR3 cells were pretreated with 1 mM DFMO for 24 h prior to treatment with 10 µM rucaparib and a 20 min pulse with 1 mM H_2_O_2_. Cells were harvested 24 h after H_2_O_2_ treatment. Lysates were probed for γH2AX and histone H3 in a Western blot assay. Figure S4: Full Western blot images for those in Figure S4A,B.
