# Supplementary material for: Difluoromethylornithine (DFMO) Enhances the Cytotoxicity of PARP Inhibition in Ovarian Cancer Cells
Source: Med Sci (Basel). 2022 May 26;10(2):28. doi: 10.3390/medsci10020028 (PMC9230675; doi:10.3390/medsci10020028)

Figure S1

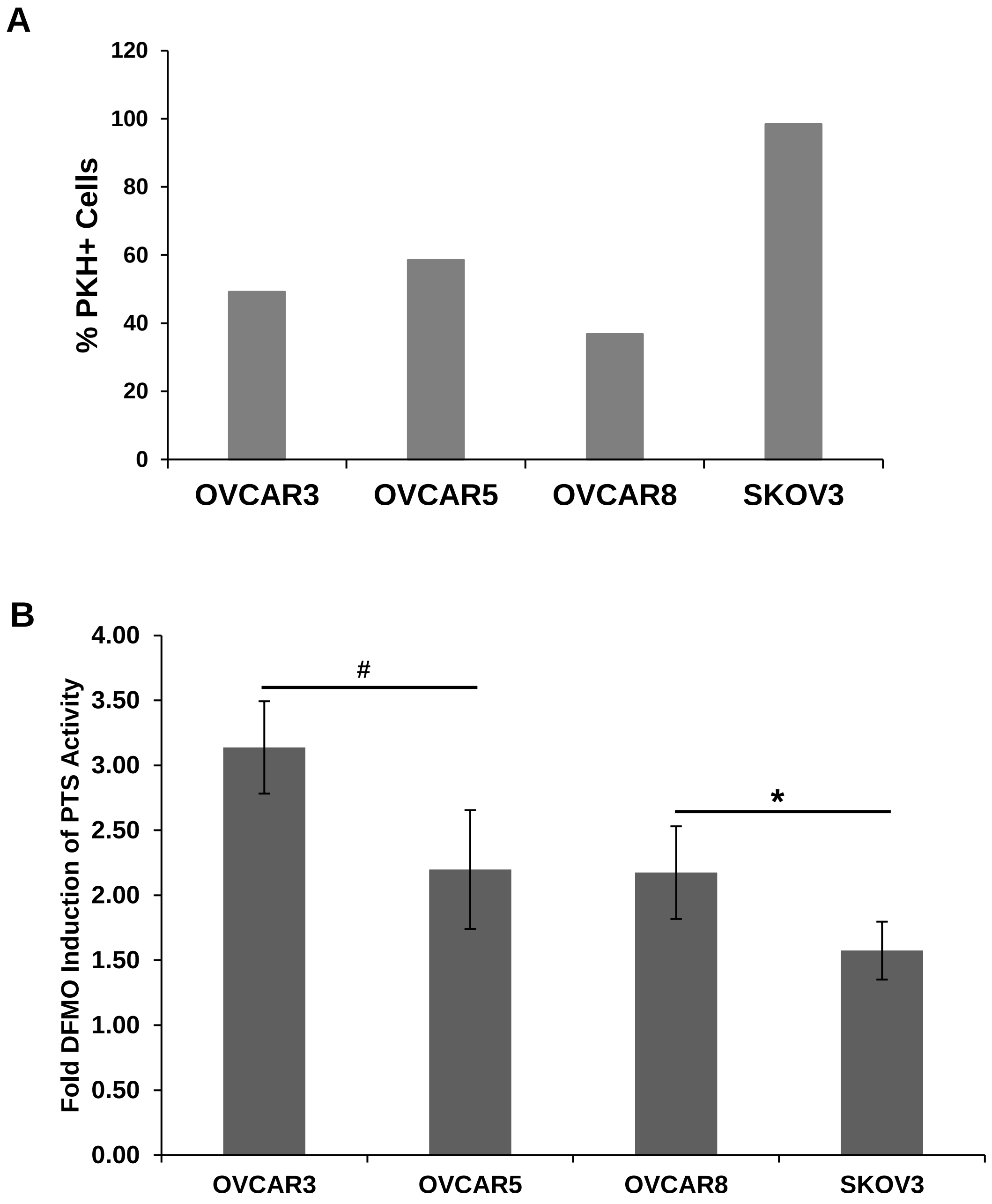
A
120
100
80
60
40
20
0
% PKH+ Cells
OVCAR3
OVCAR5
OVCAR8
SKOV3
B
4.00
3.50
3.00
2.50
2.00
1.50
1.00
0.50
0.00
Fold DFMO Induction of PTS Activity
#
*
OVCAR3
OVCAR5
OVCAR8
SKOV3

Figure S2

A

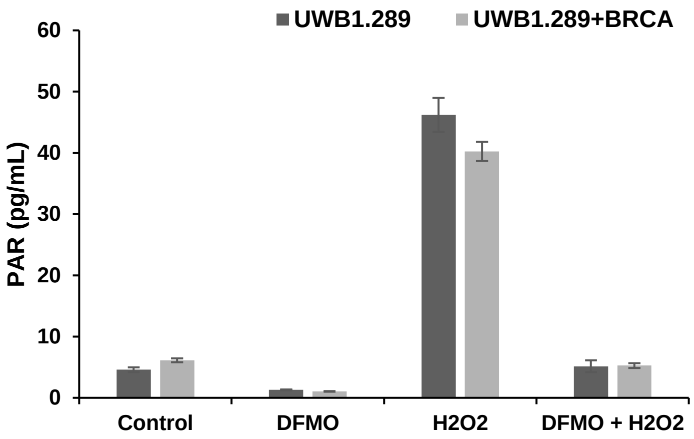
UWB1.289
UWB1.289+BRCA
PAR (pg/mL)
60
50
40
30
20
10
0
Control
DFMO
H2O2
DFMO + H2O2

Figure S3

A

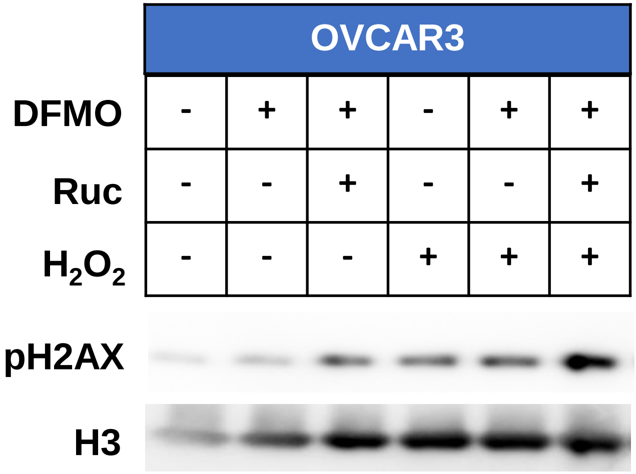

Figure S4A

Full Western blot images for Figure 4A

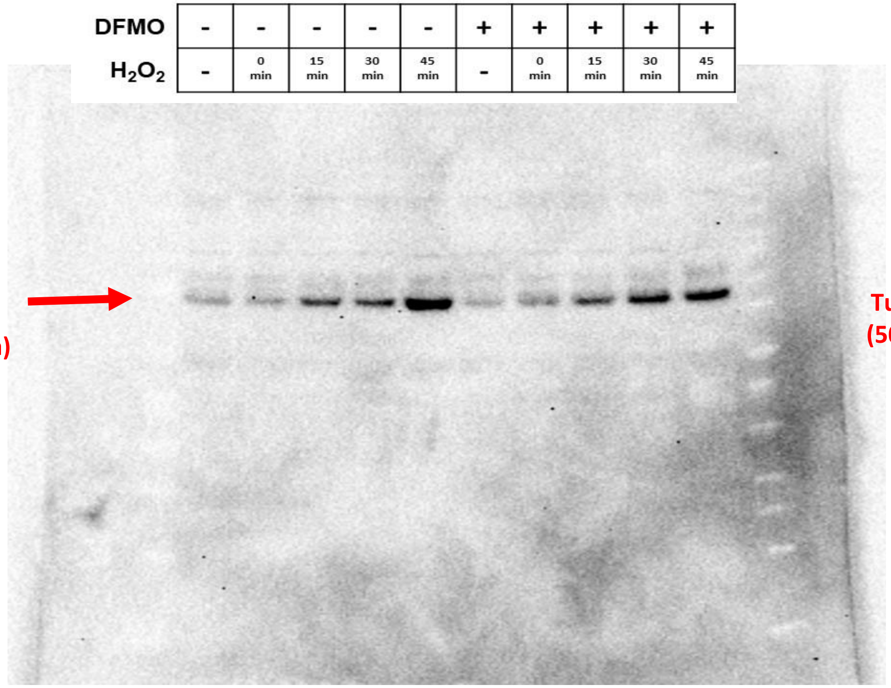

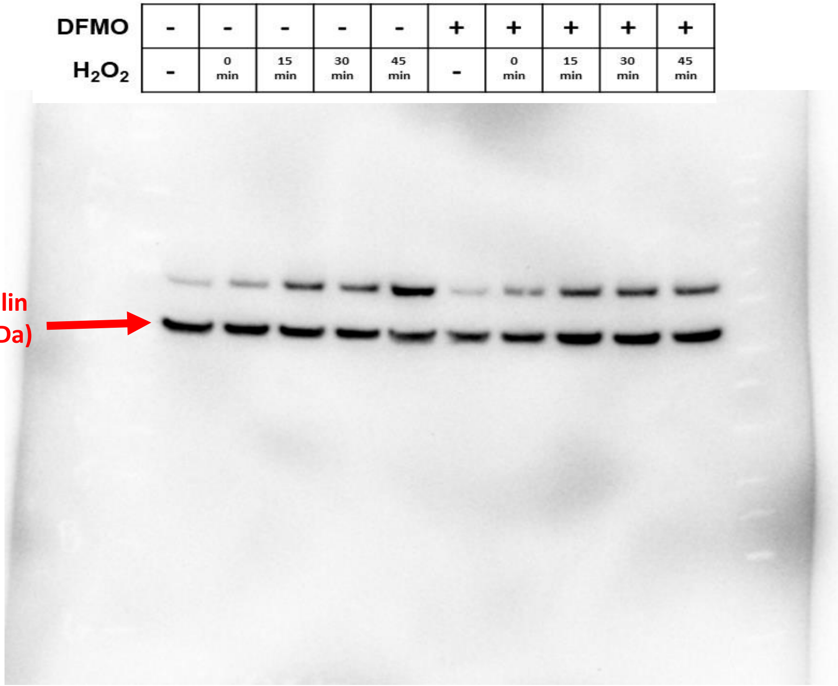

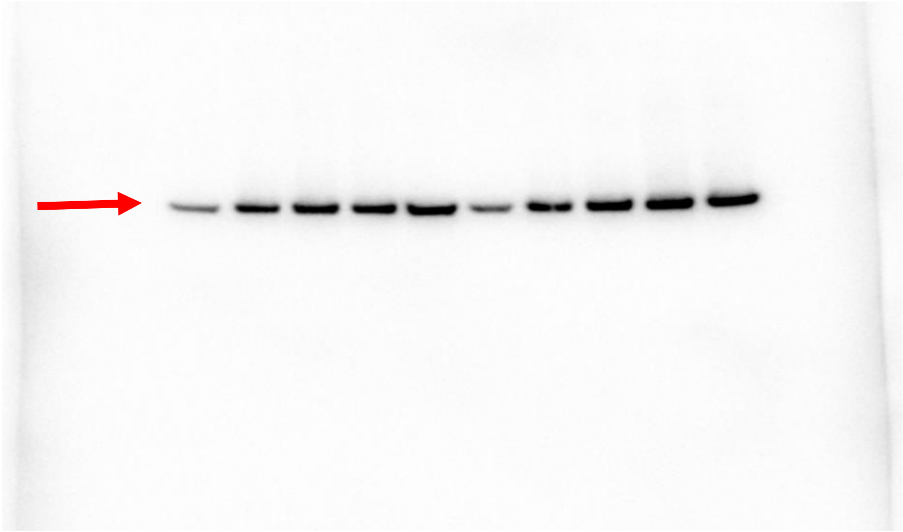

Figure S4B

Full Western blot images for Figure 4B

DFMO	-	-	-	-	-	-	-	+	+	+	+	+	+	+
H_2O_2	0	0.1 mM	0.5 mM	1.0 mM	2.0 mM	4.0 mM	10 mM	0	0.1 mM	0.5 mM	1.0 mM	2.0 mM	4.0 mM	10 mM

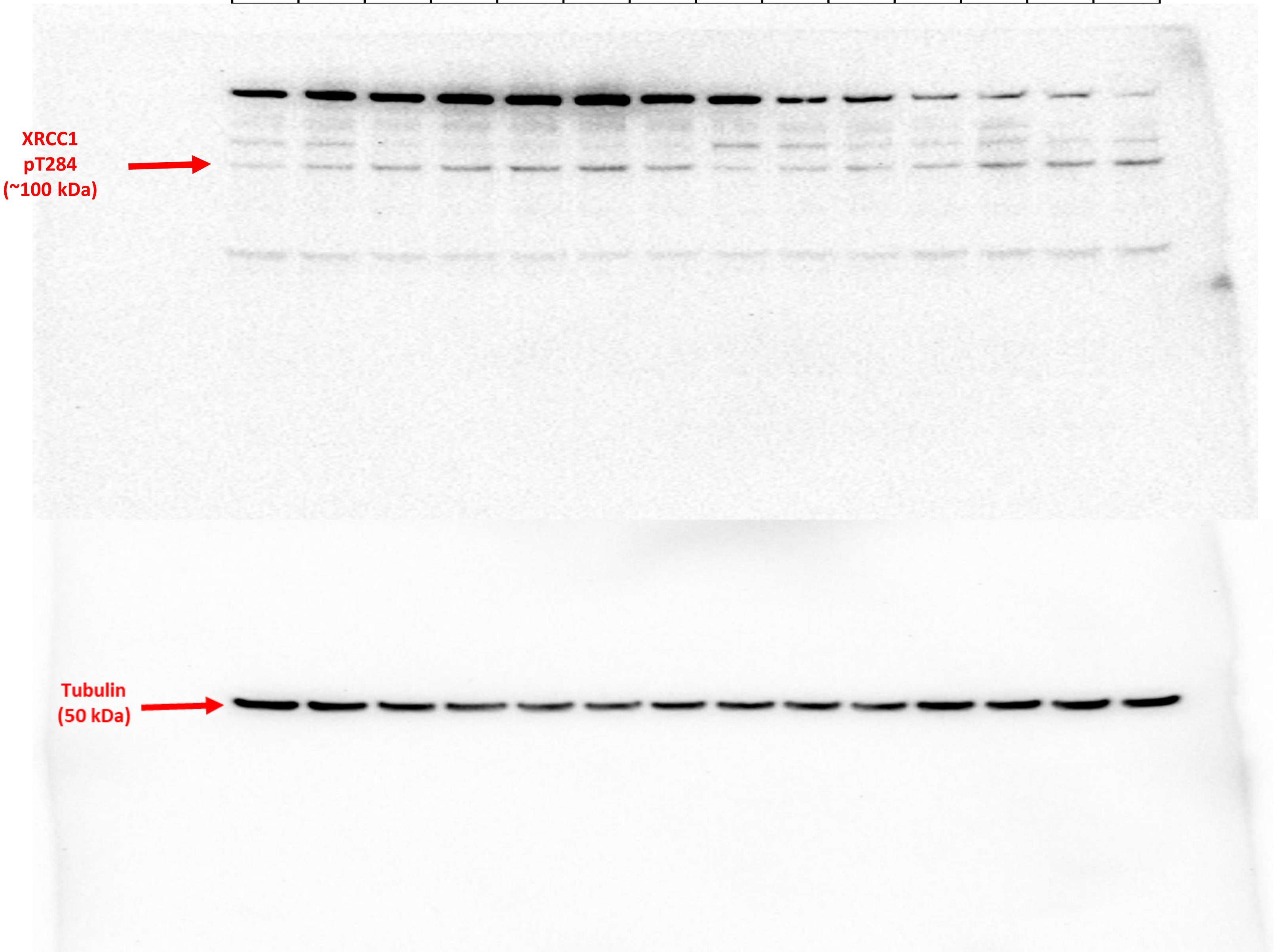

Supplement: Supplementary file 1 [file medsci-10-00028-s001.zip › medsci-1733152-supplementary.pdf]
